# Xuesaitong injection as one adjuvant treatment of acute cerebral infarction: a systematic review and meta-analysis

**DOI:** 10.1186/s12906-015-0560-4

**Published:** 2015-02-27

**Authors:** Xiaomeng Zhang, Jiarui Wu, Bing Zhang

**Affiliations:** Department of Clinical Chinese Pharmacy, School of Chinese Materia Medica, Beijing University of Chinese Medicine, Beijing, 100102 China

**Keywords:** Xuesaitong injection (XST), Acute cerebral infarction (ACI), Systematic review, Meta-analysis

## Abstract

**Background:**

Xuesaitong Injection (XST) is one of the most commonly used medicines for treating acute cerebral infarction (ACI) in China. However, compared to the conventional therapy with western medicines (WM), the effectiveness and safety of XST as an adjuvant treatment for ACI needs to be systematically reviewed.

**Methods:**

Randomized controlled trials (RCTs) comparing XST & WM with WM for treating ACI were included. Two reviewers independently extracted data. The Cochrane table of Risk of Bias was used to assess the quality of the included studies, and a meta-analysis was conducted using Review Manager 5.2.

**Results:**

23 RCTs, involving 2196 participants, were included in this study. Methodological quality was not well. The meta-analysis indicated that compared to WM, the combined use of XST and WM was more effective in terms of the total clinical effective rate [*RR* = 1.21, *95% CI* (1.16, 1.25), *P* < 0.00001], neurological deficit scores [*MD* = −3.31, *95% CI* (−4.10, −2.52), *P* < 0.00001], and plasma viscosity [*MD* = −0.13, *95% CI* (−0.15, −0.11), *P* < 0.00001]. The included studies reported 37 adverse events, 17 of which belonged to experimental groups.

**Conclusion:**

XST combined with WM appeared to be effective for ACI. However, the evidence of XST for treating ACI should be carefully interpreted due to the small sample size, limited number of trials, and unsatisfactory quality of research.

## Background

Acute cerebral infarction (ACI) is one of the most commonly seen cerebral vascular diseases, which accounting for about 70% of stroke [1]. The incidence, mortality and recurrence rate of ACI are both high, usually leads to serious damage of central nervous system [2]. ACI refers to the process that artery stenosis or blockage causing brain tissue hypoxic ischemia, and then resulting in brain dysfunction [3]. Currently, the conventional therapy with western medicines (WM) mainly includes thrombolysis, improving microcirculation, restoring blood supply to ischemic area, using cerebral protection agents, controlling cerebral edema, preventing and treating complications, controlling hypertension, reducing blood viscosity, and so on [1,2].

Some scholars hold the opinion that in traditional Chinese medicine (TCM), the pathogenesis of ACI is due to the disorder of Qi and blood, and then cerebral veins blocking, brain cells ischemia and hypoxia [4]. Therefore, the treatment of promoting blood flow is the first choice. There are numbers of literatures reported that Chinese medicinal herbs have unique advantages in the treatment of ACI by removing stasis. Xuesaitong Injection (XST), as one of the most commonly used medicines for treating ACI in China. Its active ingredient is attributed to extract from the roots of *Pannax notoginseng* (Sanqi), which mainly contains tetracyclic triterpenoid saponins, generally called as “Panax notoginseng saponins” (PNS). PNS has good therapeutic effects on cardiovascular and cerebrovascular system, blood system, and nervous system. Some pharmacological studies also showed that XST can reduce the edema caused by cerebral ischemia, protect vascular endothelial cells, inhibit the adhesion and aggregation of platelet, dissolve thrombus, eliminate superoxide anion radicals, and so on [5-9], which are all beneficial for treating acute stroke [10].

There were two systematic reviews regarding XST in the treatment of cerebral infarction [11,12], both showing the superiority of XST to control group, their delimiting of acute stage, unreasonable merging of intervention measures, and low quality of literatures still being controversial. Accordingly, in this systematic review, we chose the published, qualified and well homogeneity clinical studies regarding the combined use of XST for treating ACI to make meta-analysis, hopefully to provide more reliable evidence for XST’s clinical application.

## Methods

### Inclusion criteria

(1) Study type: clinical randomized controlled trials (RCTs) using XST as the adjuvant treatment of ACI, regardless of blinding. (2) Participants: The diagnostic criterion in terms of TCM was “Apoplexy diagnostic efficacy assessment standards”; that used in terms of WM was “various types of cerebrovascular disease diagnostic points”, as determined after 1995 [13]. Diagnoses were validated using computed tomography or magnetic resonance imaging scanning. The course of disease was in 3 days or shorter, and all participants were experiencing the first onset of ACI, no limits on age, gender, race or severity of disease. (3) Interventions: RCTs comparing combined therapy of XST and conventional treatments versus conventional treatments alone were included. Conventional treatments included thrombolytic therapy, cerebral protection agents, and so on. The drugs could be Dextran-40, Mannitol, Aspirin, Citicoline, Venoruton, Defibrase, Sodium ozagrel, et al. As long as the conventional treatments were the same between study groups in one RCT, the RCT could be included. No limitation on the dosages and treatment courses. (4) Outcomes: Our primary outcome measure was the total clinical effective rate, using the following formula: total clinical effective rate (%) = (number of recovered patients + number of patients with significant progress + number of patients with progress) / total number × 100%. Efficacy criteria predominantly referred to the reduction of neurological deficit score. Recovered was determined when the neurological deficit score decreased from 91% to 100%. Significant progress was determined when the neurological deficit score decreased by between 46% and 90%. Progress was determined when the neurological deficit score decreased by between 18% and 45%. No change or worsen was determined when the functional deficit score decreased by <17%. As secondary outcomes, we compared neurological deficit score, hemorheology value, and number of adverse drug reactions (ADR)/adverse events (AE).

### Exclusion criteria

If involved any condition of the followings, trials can be ruled out: data was incorrect, incomplete or not available; patients with severe cognitive disorder, or hemorrhagic tendency, or serious complications, such as atrial fibrillation, severe heart failure, severe liver and kidney diseases; undergoing surgery, acupuncture or other physical therapy; combined with any herbal medicines in experimental or control group during the treatment.

### Searching strategies

The following databases were used for search: the China National Knowledge Infrastructure Database (CNKI, 980–2014.5), Wan fang Database (1990–2014.5), China Science and Technology Journal Database (VIP, 1989–2014.5), Chinese Biomedical Literature Database (CBM, 1981–2014.5), PubMed (1990–2014.5), Embase (1990–2014.5), and the Cochrane Library (1990–2014.5).

We combined different search strategies as follows: for English databases, we used free text terms as “Xuesaitong” and “acute cerebral infarction”; for Chinese databases, we used subject terms as “Xuesaitong Zhu She Ye” or “Zhu She Yong Xuesaitong ”or “Xuesaitong Dong Gan Fen Zhen” or “Luotai”, and then “Que Xue Xing Cu Zhong” or “Que Xue Xing Zhong Feng” or “Que Xue Xing Nao Xue Guan Bing” or “Nao Geng Si” or “Nao Geng Se” for secondary retrieval. No language restriction was used.

### Data extraction and quality assessment

Two reviewers (XZ and JW) independently screened trials. If there was incomplete information in the study, the reviewer would firstly contact original authors via e-mail or telephone. If no response, the reviewer would make decision on including or excluding based on the importance of lack information. XZ and JW independently extracted data, including basic information of patients, interventions, duration of treatment, outcomes and methodological quality. And the risk of bias of the included trials was assessed according to the Cochrane risk of bias tool [14]. It assessed the risk of bias of random sequence generation, allocation concealment, blinding of participants and personnel, blinding of outcome assessment, incomplete outcome data, selective reporting, and other biases. Judgments were given for each item as: low, unclear, and high risk of bias. XZ and JW independently completed and mutually checked the allocated grades. Any disagreements on data extraction and quality assessment were resolved by consensus, or if required by a third reviewer.

### Data synthesis

RevMan 5.2 software was used, whose package was produced and updated by the Nordic Cochrane Centre. Relative risk (*RR*) was used for dichotomous data, and mean difference (*MD*) was used for continuous variables, both with 95% confidence interval (*95% CI*), *P* < 0.05 was considered statistically significant between experiment and control group. The Chi-square test was used for checking the heterogeneity between studies, and *I*^*2*^ was used to show the size of heterogeneity. If *P* > 0.1 and *I*^*2*^ < 50%, there was determined to be little heterogeneity between studies, then we used a fixed effect model, otherwise we should use a random effect model. If the number of included trials was sufficient, a funnel plot would be carried out to assess publication bias. Sensitivity analysis was performed to inspect the robustness of the result.

## Results

### Searching result

**Figure 1:**
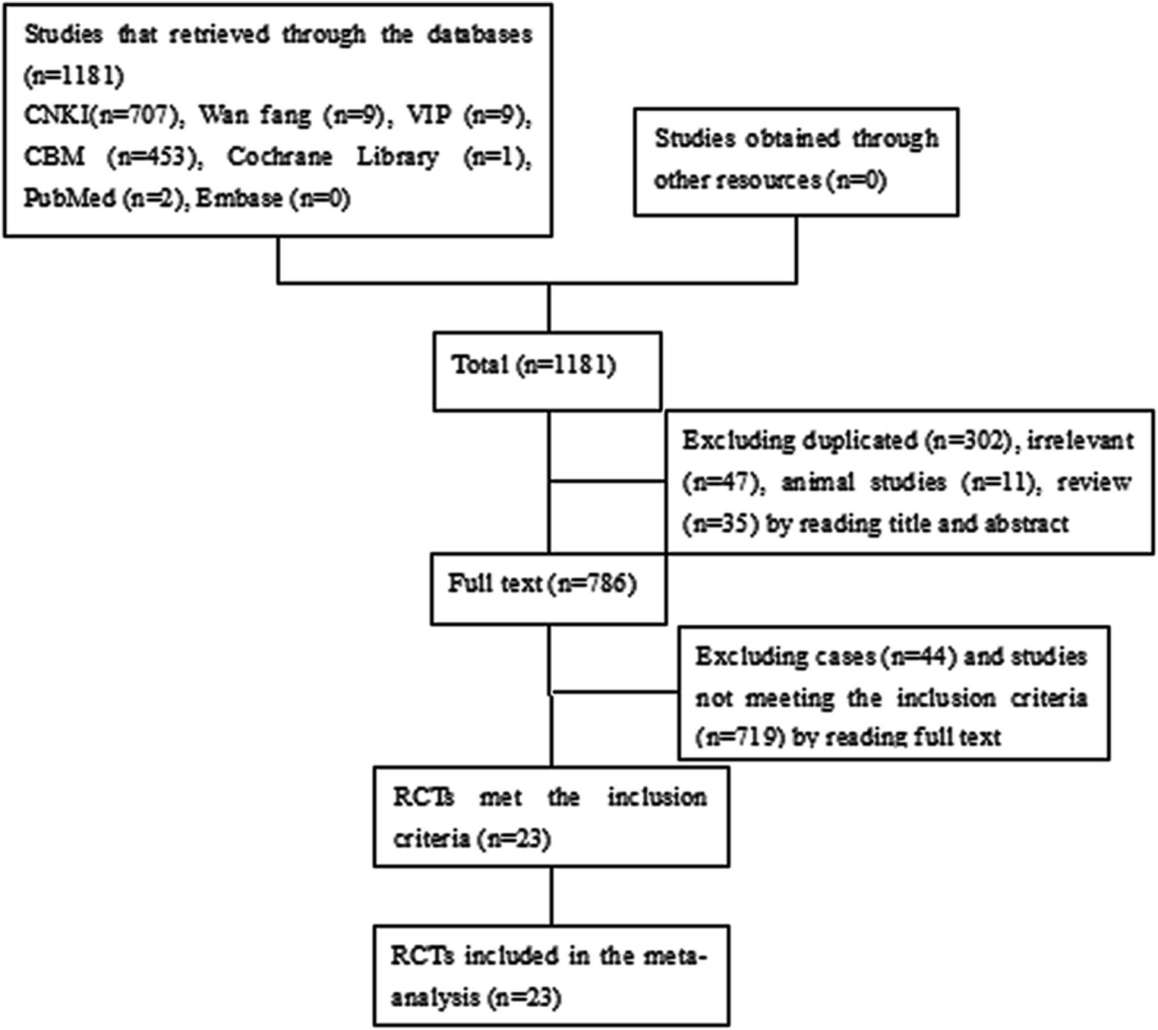
**Flow chart of literature search.**

Total 1181 articles were retrieved from the databases listed above. Finally, a total of 23 RCTs were included [15-37] (Figure 1 flow chart of literature search).

### Studies description

The included studies were published between 1999 and 2013. All RCTs originated from China, and were published in Chinese. Altogether, 23 RCTs included a total of 2196 patients. As presented in Table 1, the experimental group consisted of 1141 patients, while the control group 1055. The included patients were primarily elderly, the average age of which was about 60.5, with a range of 33 to 86 years old. 58.4% of the participants were males. In the experimental group, all the RCTs used XST combined with the same WM as control group. The daily dose of XST ranged from 200 ~ 600 mg. In the included RCTs, XST was produced by Xing Zhong Pharmaceutical Co., Ltd. in Kunming, Plant Pharmaceutical Co., Ltd. in Yunnan Province, China, or Zhenbaodao Pharmaceutical Co., Ltd. in Heilongjiang Province, China. The duration of treatments ranged from 14 days to 28 days. More details were shown in Table 1.

**Table 1:** **Characteristics of included trials on XST for ACI**

**Study ID**	**Sex (M/F)**	**Age (a)**	**Course of disease (h)**	**N (E/C)**	**Intervention**		**Duration (days)**	**Outcomes**	**ADR/ADE**
					**Experimental**	**Control**			
Li 1999 [15]	29/33	62.9 ± 7.1 (57 ~ 71)	≤48	31/31	XST 400 mg + WM	WM (Defibrase et al.)	14	Total effective rate, changes in cerebral blood flow and hemorheology	Unclear
Zhang 2002 [16]	52/48	53.0 (37 ~ 70)	≤72	60/40	Luotai 400 mg + WM	Xiaoshuanling + WM	14	Total effective rate	Unclear
Yuan 2003 [17]	37/25	42 ~ 79	≤72	32/30	XST 20 ml + WM	WM (Piracetam et al.)	15	Total effective rate, changes in hemorheology	Unclear
Li 2003 [18]	69/27	55.8 (42 ~ 71)	≤72	48/48	XST 400 mg	WM (Dextran 40 + citicoline)	15	Total effective rate	Unclear
Li 2005 [19]	-	48 ~ 73	≤72	80/76	XST 200 mg + WM	WM	20	Total effective rate	E 3
He 2006 [20]	48/32	59.3 ± 12.1 (46 ~ 80)	≤72	40/40	XST 10 ml + WM	Dextran 40 + WM	14	Total effective rate, neurological deficit score	None
Wang 2006 [21]	34/21	63.8 (39 ~ 79)	≤24	36/19	XST 600 mg + WM	Venoruton + WM	21	Total effective rate	None
Yuan 2006 [22]	64/31	63.0 ± 10.4 (46 ~ 74)	≤24	49/46	XST 250 ~ 500 mg + WM	WM (Defibrase et al.)	15	Total effective rate	E 3
									C 7
Zhao 2006 [23]	43/38	60.9 ± 8.1 (45 ~ 70)	≤72	40/41	XST 10 ml + WM	WM (Sodium ozagrel + Defibrase et al.)	14	Total effective rate, changes in hemorheology	Unclear
Li 2007 [24]	54/36	59.5 ± 13.2 (48 ~ 79)	≤48	45/45	XST 10 ml + WM	Dextran 40 + WM	15	Total effective rate	None
Wang 2007A [25]	50/26	63.6 (39 ~ 82)	≤24	50/26	XST 600 mg + WM	Venoruton + WM	21	Total effective rate	None
Wang 2007B [26]	-	69.4 (43 ~ 80)	≤72	30/30	XST 20 ml + WM	WM (Venoruton et al.)	14	Total effective rate	None
Rong 2008 [27]	50/46	59.1 ± 8.4 (39 ~ 77)	≤72	51/45	XST 400 mg + WM	Venoruton + WM	14	Total effective rate, neurological deficit score, Modified Barthel Index, changes in hemorheology	None
Zhang 2008 [28]	70/50	56 ± 11 (48 ~ 78)	≤48	65/65	XST 200 ~ 400 mg + WM	WM	15	Total effective rate, changes in hemorheology	Unclear
Zi 2008 [29]	49/33	59.5 ± 13.2 (48 ~ 79)	≤48	41/41	XST 10 ml + WM	Dextran 40 + WM	15	Total effective rate, neurological deficit score	None
Duan 2009 [30]	35/34	64.5 ± 8.2 (33 ~ 75)	≤48	36/33	XST 500 mg + WM	WM	14	Total effective rate	None
Ma 2009 [31]	117/83	65.1 ± 7.0 (45 ~ 85)	≤72	100/100	XST 400 mg + WM	Venoruton + WM	15	Total effective rate, neurological deficit score, changes in hemorheology, glycemia and lipidemia	E 9
									C 10
Cai 2011 [32]	42/18	64.2 (47 ~ 86)	≤48	30/30	XST 400 mg + WM	WM (Buflomedil hydrochloride + Low molecular heparin)	14	Total effective rate, neurological deficit score	E 2
									C 1
Fu 2011 [33]	62/58	55.4 ± 5.1	≤48	64/58	XST 400 mg + WM	WM (Sodium ozagrel et al.)	14	Total effective rate, neurological deficit score	None
He 2011 [34]	57/65	59 ~ 78	≤72	62/60	XST 200 mg + WM	WM (Citicoline, Aspirin)	14	Total effective rate, neurological deficit score	None
Wang 2011 [35]	45/37	55.87 ± 5.23 (42 ~ 76)	≤19	41/41	XST 400 mg + WM	WM	21	Total effective rate, Hs-CRP	Unclear
Yang 2012 [36]	33/27	57 ± 4 (47 ~ 68)	≤48	30/30	XST 400 mg + WM	WM (Sodium ozagrel et al.)	14	Total effective rate	C 2
Song 2013 [37]	117/43	62.1	≤24	80/80	XST 400 mg + WM	WM	15	Total effective rate	Unclear

NOTE: M: Males; F: Females; E: Experimental group; C: Control group; ADR: Adverse drug reactions; ADE: Adverse drug events; XST: Xuesaitong Injection; WM: Conventional therapy with western medicines, such as Dextran-40, Mannitol, Aspirin, Citicoline, Venoruton, Defibrase, Sodium ozagrel, et al.

### Quality of the included studies

**Figure 2:**
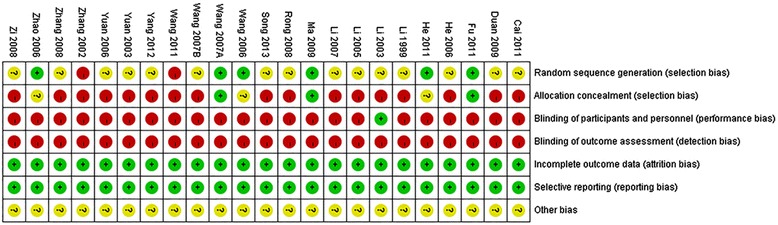
**Risk of bias.**

**Figure 3:**
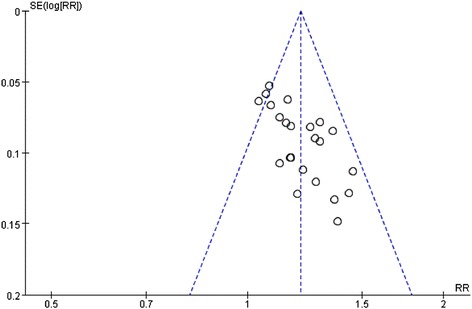
**Funnel plot of the total effective rate of XST in the treatment of ACI.** XST: Xuesaitong Injection; ACI: acute cerebral infarction.

Three trials used a random number table to generate random sequence [26,32,34], two grouped according to the time of admission [21,35], and one grouped according to odd and even numbers [24]. And there were two trials may be used non-random sequence [16,36]. Only one trail performed single-blinding [18]. Others in the included trials in this meta-analysis were judged as unclear random sequence generation, inadequate allocation concealment, and inadequate blinding. None of the included trials had incomplete outcome data or selective reporting. Therefore, the quality of the included studies was not well. More details of the trials were presented in Figure 2. Funnel plot analysis showed that there was some significant publication bias in the comparison, as shown in Figure 3.

### Effects of interventions

#### Total clinical effective rate

**Figure 4:**
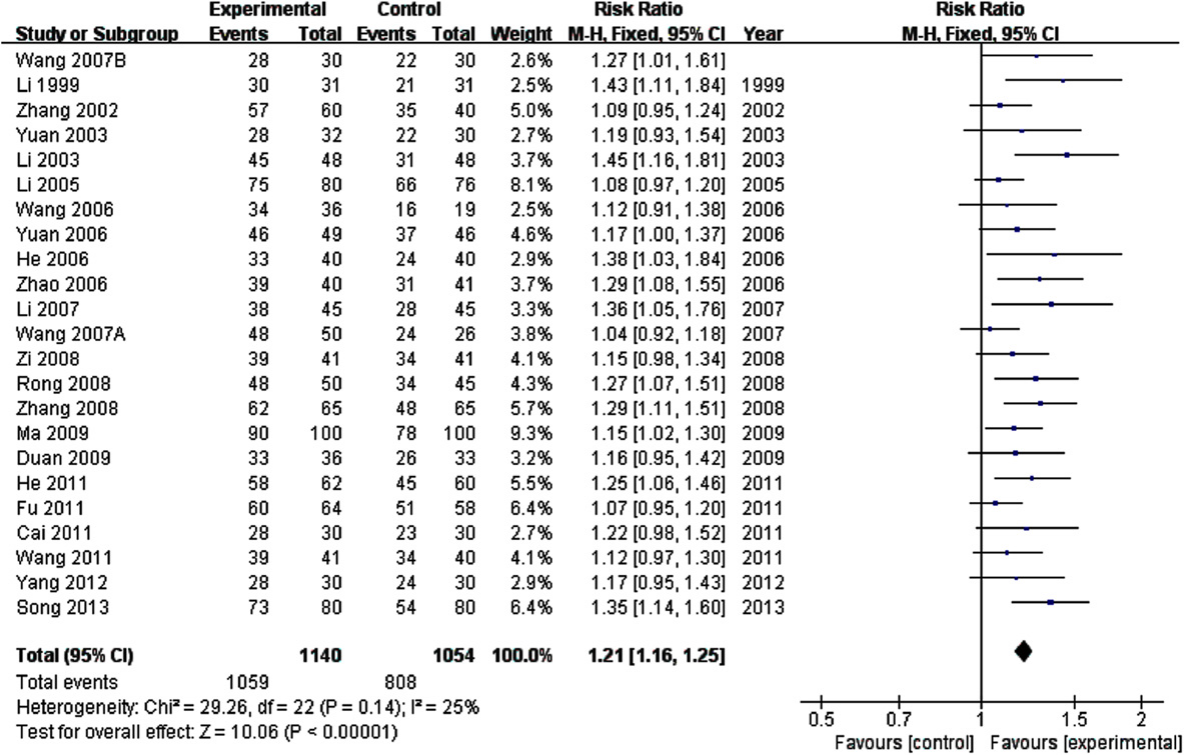
**Meta-analysis of the total effective rate of XST in the treatment of ACI.** XST: Xuesaitong Injection; ACI: acute cerebral infarction.

All the included studies [15-37] reported the total effective rate. The meta-analysis showed that the effect of XST combined with WM was better than WM alone in improving the total effective rate. The statistical difference between the two groups was significant [23RCTs, *I*^*2*^ = 25%, fixed effect model, *RR* = 1.21, *95% CI* (1.16, 1.25), *P* < 0.00001]. (As shown in Figure 4).

### Sensitivity analysis

To confirm the stability of the result of the total clinical effective rate, we respectively removed the most and the least weighted, and changed from fixed mode to random mode. After removing the most weighted (Ma et al. [31]), the result was *RR* = 1.21 [*95% CI* (1.17, 1.26), *P* < 0.00001]. The result of removing the least (Li et al. [15] and Wang et al. [21]) was *RR* = 1.20 [*95% CI* (1.16, 1.25), *P* < 0.00001]. The result of changing the mode was *RR* = 1.18 [*95% CI* (1.13, 1.23), *P* < 0.00001]. There was no obviously diversity, so the degree of the sensitivity of the study was not high.

### Neurological deficit score

**Figure 5:**
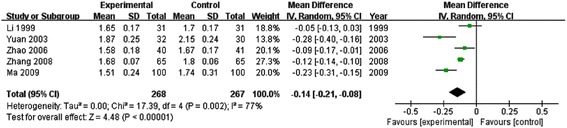
**Meta-analysis of reducing neurological deficit scores of XST for testing ACI.** XST: Xuesaitong Injection; ACI: acute cerebral infarction.

There were seven trials mentioned neurological deficit score [20,27,28,31-34]. Heterogeneity between studies was large (*P* < 0.00001, *I*^*2*^ = 84 > 25%), we should use a random effect model. The result showed that XST was more effective in reducing neurological deficit score. The statistical difference between the two groups was significant [*MD* = −4.35, *95% CI* (−6.61, −2.08), *P* = 0.0002]. (Shown in Figure 5).

### Plasma viscosity

**Figure 6:**
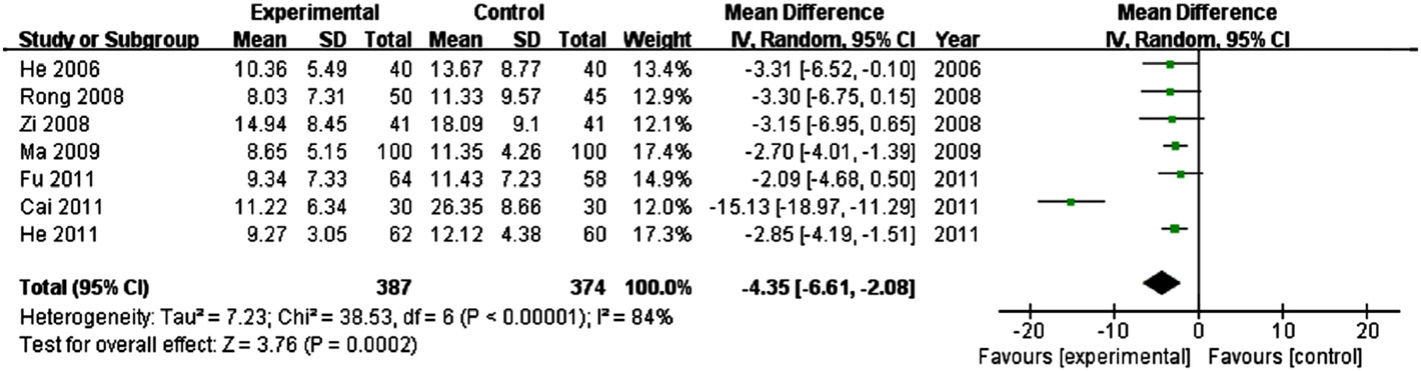
**Meta-analysis of reducing the content of plasma viscosity of XST for treating ACI.** XST: Xuesaitong Injection; ACI: acute cerebral infarction.

There were five trials mentioned the content of plasma viscosity [15,17,23,28,31]. The result showed that XST was more effective in reducing the content of plasma viscosity. The statistical difference between two groups was significant [5 RCTs, *I*^*2*^ = 77%, random effect model, *MD* = −0.14, *95% CI* (−0.21, −0.08), *P* < 0.00001]. (Shown in Figure 6).

### Adverse events

Five trials [19,21,32,33,37] reported 35 cases of AE. None of participants who reported AE developed bleeding or liver and kidney damage, and all participants with AE recovered after symptomatic treatments. The occurrence of AE did not affect treatment process. There were 17 from 35 cases occurred in experimental group, which were manifested as two cases of headache and nausea, three cases of rash, three cases of a small amount of bleeding points in subcutaneous, four cases of dizziness, palpitation, and five cases of flush. While 20 cases occurred in control group, which were manifested as one case of bleeding gums, one case of gastrointestinal bleeding, three cases of nausea and vomiting, four cases of dizziness and palpitation, five cases of bleeding points in subcutaneous, and six cases of face red. Ten trials clearly expressed that no ADRs/ADEs occurred in their studies [20,21,25-28,30,31,34,35]. And the other eight trials provided no information.

## Discussion

### Discussion on the systematic review

Following the systematic review, we can summarize that XST as an adjuvant treatment for WM for ACI was more effective. It could increase the total clinical effective rate, decrease the degree of neurological deficit, and reduce the content of plasma viscosity, which were consistent with results of previous system reviews [11,12]. Compared with previously reported data, we were not only adding more high-quality, small heterogeneous literatures, but also strictly controlling the course of disease and the medications of control group. Therefore, the present systematic review was more scientific and precise. But regarding outcome of neurological deficit scores, Cai 2011 [32] reported large heterogeneity with the other included studies, which may be related to less effectiveness of Buflomedil Hydrochloride and low molecular heparin on improving neurological deficit. And though the efficacy of XST for treating ACI is sure, its safety remains to be further assessed. Accordingly, from the existing clinical evidence, XST can be promoted using in clinical to form an integrative therapy.

### Recommendation on the efficacy evaluation of XST in the treatment of ACI

A research from Asia Pacific Region found that significant correlation exists between dyslipidemia and occurrence of ischemic stroke. When the total cholesterol (TC) increased by 1 mmol/L, the incidence of stroke will increase by 25% [38-40]. Meanwhile, experiments demonstrated that PNS, the main ingredient in XST, can significantly reduce blood lipids and lipid peroxidation products [41]. In the systematic review, only one study [31] monitored the changes in TC was *MD* = −1.01[*95% CI* (−1.41, −0.61), *P* < 0.00001)], which had significant differences between two groups. Accordingly, depending on the GRADE methods, we propose that blood lipids level should be used as one of the important indicators for the evaluation of efficacy of XST for treating ACI in the further clinical studies.

On the other hand, high C-reactive protein (Hs-CRP), as a kind of acute phase proteins, may increase rapidly when suffering various acute inflammations, tissue damage, and other diseases. When patients improved, the content of Hs-CRP quickly returned to normal. Its increasing has positive correlation with the degree of infection, and it was with high sensitivity [42]. Accordingly, Hs-CRP can be as an independent risk factor for cerebral infarction to predict the severity of cerebral infarction and infarct size. But in the included studies, only 1 trail [35] compared the changes in Hs-CRP, which result was *MD* = −6.62 [*95% CI* (−8.29, −4.95), *P* < 0.00001]. In sum, depending on the GRADE methods, we considered Hs-CRP can also serve as one of important indicators in evaluation of efficacy of ACI.

### XST’ADRs

In clinic, ADR of XST’s was reported in large quantity. The common symptoms included headache, dry throat, palpitation, pruritus, severe rash, and the severe even have anaphylactic shock [43], which are generally in consistent with the AEs that reported in our meta-analysis. Although there were no serious ADRs, we could not conclude on its safety yet. Therefore, special attention should be paid to the observation of AE. The meta-analysis showed that there were four studies [21,22,25,30], in which their dose of XST exceeded 200 ~ 400 mg, and such high dose may easily cause ADRs according to instruction manual. In addition, there were also one study [44] showed that allergies occurrence time was mostly to exceed 7d. It prompts that medical staffs need to have a properly assessment on efficacy to avoid overdose. Patients with allergic history have more chance to suffer ADR, so the clinicians need to know well the allergic history of patients. Furthermore, drug combination is also a high risk for ADR occurrence; accordingly, it should reduce the quantity of drugs as few as possible for combination use.

### Limitation of this systematic review and direction for further clinical research

Although the systematic review showed that XST as adjuvant treatment for WM for ACI was effective, the methodological quality of included studies was not ideal. Only 6 studies described random allocation method, and none of them described allocation concealment. Only one study used single-blinding. All the RCTs had small sample size. In the included studies, XST was products from two manufacturers and the dosage of XST varied a lot. In addition, the systematic review itself also has some limitations. Since all trials were published in Chinese, we could not exclude the potential publication bias. More rigorously designed RCTs are needed to confirm the effectiveness of XST for ACI.

## Conclusion

According to low quality evidences, XST as an adjuvant treatment for conventional treatments for treating ACI was beneficial comparing to conventional treatments alone. Although the safety of XST requires further research, it is worthy to be promoted using in clinical.

## Abbreviations

ACI: Acute cerebral infarction
XST: Xuesaitong injection
WM: Conventional treatment with western medicine
TCM: Traditional Chinese medicine
RCTs: Randomized controlled trials
CNKI: China national knowledge infrastructure
VIP: China science and technology journal database
CBM: Chinese biomedical literature database
ADRs: Adverse drug reactions
ADEs: Adverse drug events
RR: Relative risk
MD: Mean difference
95% CI: 95% confidence interval
